# 

**Published:** 1853-01-01

**Authors:** 


### THE JOURNAL

OF

### PSYCHOLOGICAL MEDICINE

AND

### MENTAL PATHOLOGY.

EDITED BY

FORBES WINSLOW, M.D. D.C.L.

PRESIDENT OF THE MEDICAL SOCIETY OF LONDON.

VOL. VI.

22150 Washington, D.C.

LONDON:

JOHN CHURCHILL, PRINCES STREET, SOHO.

MDCCCLIII.

W
1
JO 857 N

LONDON:
SAVILL AND EDWARDS, PRINTERS, CHANDOS STREET,
COVENT GARDEN.

We must defer our miscellaneous notices of books, both foreign and domestic, letters of correspondents, translations, and legal notes of lunacy cases, until our next number. The unexpected length of the article on "British Lunatic Asylums" has compelled us to set aside the lecture on "Mental Dynamics," and many other valuable contributions intended for this number.

